# Prognostic significance of frailty status in patients with primary lung cancer

**DOI:** 10.1186/s12877-023-03765-w

**Published:** 2023-01-25

**Authors:** Kai Wang, Quan She, Min Li, Hongye Zhao, Weihong Zhao, Bo Chen, Jianqing Wu

**Affiliations:** 1 Jiangsu Provincial Key Laboratory of Geriatrics, Department of Geriatrics, The First Affiliated Hospital of Nanjing Medical University, 300 Guangzhou Road, Nanjing, Jiangsu 210029 People’s Republic of China; 2 Department of General Practice, The First People’s Hospital of Lianyungang, Lianyungang Clinical College of Nanjing Medical Unversity, Lianyungang, 222000 China

**Keywords:** Frailty, Lung cancer, The frailty index based on laboratory test (FI-LAB), All-cause mortality risk

## Abstract

Lung cancer has one of the highest morbidity and mortality rates in the world. Frailty is common in many countries and is a major cause of premature functional decline and premature death in older adults, and may affect the treatment and prognosis of lung cancer patients. To investigate the predictive value of frailty at diagnosis on all-cause mortality in lung cancer patients, this study retrospectively collected and analysed clinical information on lung cancer patients from 2015–2018. A total of 1667 patients with primary lung cancer were finally included in this study. The median follow-up time of patients was 650 (493, 1001.5) days. A total of 297(17.8%) patients had FI-LAB(the frailty index based on laboratory test) status of frail at the moment of diagnosis and the all-cause mortality rate for all patients was 61.1% (1018/1667). In a univariate model, we found a higher total all-cause mortality risk in frail patients (frail vs. robust, HR(hazard ratio) = 1.616, 95% CI(confidence interval) = 1.349,1.936), after balancing other variables combined into model 1 to model 6. The results were analyzed visually using ROC(Receiver operating characteristic) curves with nomogram and the AUC values ranged from 0.866–0.874. The final inclusion of age, TNM stage, CCI(Charlson comorbidity index) score, surgery history and chemotherapy into a multifactorial model balanced the predictive power of frailty grading on all-cause mortality. The study showed that for lung cancer patients, the higher the level of frailty at diagnosis, the higher the risk of all-cause mortality. In the context of widespread electronic medical records in hospitals, it is convenient and feasible to use FI-LAB to assess the prognosis of lung cancer patients.

**Supplementary Information:**

The online version contains supplementary material available at 10.1186/s12877-023-03765-w.

## Introduction

Lung cancer has one of the highest morbidity and mortality rates in the world [1]. More than half of patients with a first diagnosis of lung cancer are older than 70 years [2]. Previous studies have found that patients' own factors (age, sarcopenia, more comorbidities) and tumor-related factors (pathological staging, TNM stage) affect patient prognosis [2–4]. Comprehensive geriatric assessment prior to treatment is a multidimensional assessment of health status in physical, functional, and psychosocial domains, and studies have shown that geriatric impairment and frailty have a high predictive value for survival [5].

Frailty is a complex clinical syndrome caused by a decline in the reserve and function of multiple physiological systems [6, 7], which is one of the leading causes of premature frailty and premature death in the elderly [8]. The probability of frailty is increasing in healthy older people over 70 years of age, over 25% in those over 85 years old [9, 10]. Some studies had emphasized the assessment of frailty in tumor patients [11, 12]. However, individualised treatment protocols for tumour patients based on the degree of debilitation are still being explored, geriatric assessment (GA) is the most appropriate solution to this dilemma [11, 12]. There is a growing body of research on GA assessment [7]. Screening often used as the first step in frailty management [13]. Geriatric-8 and Vulnerable Elders are the most commonly used as initial screening tools [14, 15]. The next step was to apply clinical judgement to identify the degree of frailty [16]. Additionally, as summarized in the studies of She et al., the clinical frailty scale (CFS), FI (frailty index), frailty phenotype, frail scale, and Edmonton Frailty Scale (EFS) had been shown to assess frailty. However, they were more difficult to generalize due to the subjective nature of their assessment scales and poor data availability [8, 17–20]. Fan demonstrated that FI was associated with all-cause specific mortality in young and elderly Chinese [21]. The frailty index based on laboratory test (FI-LAB), defined as the proportion of aberrant results from the total of measured tests [22, 23]. FI-LAB calculated from laboratory tests can quickly and effectively screen the frailty [7]. The initial screening allows early detection of frailty and risk of frailty, which improves the patient's overall awareness of frailty [24].

To date, no consistent conclusions have been reached in studies related to FI and all-cause mortality in lung cancer patients. Based on retrospective data from the medical record system of First Affiliated Hospital with Nanjing Medical University (NJMU) from 2015–2018, this study intends to investigate the value of FI-LAB in predicting survival and all-cause mortality in lung cancer patients after adjusting for other factors.

## Methods

### Data sources and study population

This study utilized the electronic medical record system of the Department of Geriatrics, First Affiliated Hospital of Nanjing Medical University (NJMU) from 2015–2018 for retrospective analysis. The data was extracted independently by two researchers and cross-checked. Inclusion criteria were as follows: 1. diagnosed with primary lung cancer; 2. age 18 years or older; and 3. verbal consent to anonymize data during the telephone follow-up was included in this study. Exclusion criteria: 1. Patients who were lost to telephone or outpatient follow-up; 2. patients with carcinoma in situ (stage 0); 3. patients with other tumors metastasizing to the lung; 4. patients for whom baseline data or frailty score data were missing. Informed consent for telephone follow-up in this study was provided verbally by all successfully followed subjects, as it was not feasible to obtain written consent during telephone follow-up, which was also approved by the Ethics Committee of the First Affiliated Hospital of NJMU (2021-SR-243). This study was conducted in accordance with the Declaration of Helsinki.

### FI-LAB definition

The FI-LAB values were calculated using the cumulative deficit model developed at the West China Hospital, which was constructed from laboratory test of the baseline deficiency status of the seven systems: routine blood tests, Hepatic Function, Fast Blood Glucose, Renal Function, Blood Lipid, Blood Electrolyte, Blood Coagulation^25^. We extracted 44 health-related variables from the medical record data. Each variable is defined using a binary indicator of 0 and 1, with 0 indicating that the test indicator is then within the normal range of values. The higher the FI-LAB value, the higher the degree of frailty of the patient. According to the study of Wang et al. [25], we used 0.2 versus 0.35 as a cut-off to distinguish between robust, pre-frail and frail.

### Follow-up and clinical outcomes

The main focus of this study was the survival time of the patients. Information on patient deaths included deaths during hospitalization and telephone follow-up after patient discharge. In this study, a total of 2 investigators conducted telephone follow-up, and information on patients' treatment and death events was obtained from interviews and checked with treatment information in our hospital database. Survival time for this study was defined as initial diagnosis to death or last follow-up visit.

### Data analysis

For normally distributed continuous variables, we used the mean and standard deviation; for non-normally distributed continuous variables, the median and quartiles were used. For binary and categorical variables, we used numbers and percentages to describe the population. The t-test was used to analyze differences between groups, and we defined patients with FI-LAB < 0.2 as robust, FI-LAB 0.2–0.35 as pre-frail, and those with FI-LAB > 0.35 were defined as frail. For categorical variables, the card method was used to analyze differences between two groups, and for continuous variables, variables with normality and homogeneity of variance were analyzed using the Student's t test and ANOVA to select variables with significant significance (p < 0.05) for additional analysis and to visualize the relative results of the analysis of variance. We then developed a multivariate Cox regression model using the variables with significant differences defined in the univariate analysis. The results of the regression analysis were expressed as dominance ratios (ORs) and 95% confidence intervals (CI). Receiver operating characteristic (ROC) curves were plotted for model evaluation, and column line plot analysis was performed to describe the variable scores for risk prediction. All statistical analyses were performed in R software version 4.0.5.

## Results

### Baseline clinical characteristics

**Fig. 1:**
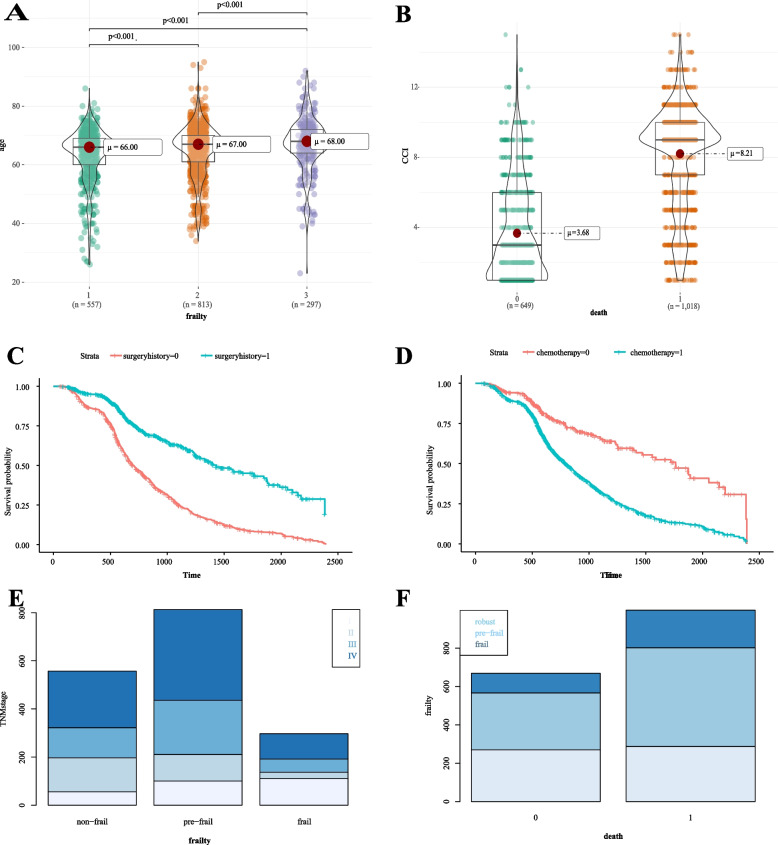
Baseline clinical characteristics. **A** indicates in the robust, pre-frail, and frail groups; **B** indicates the CCI distribution in the survival and death groups; **C** and **D** reflect the survival differences among patients with different surgical and chemotherapy histories; **E** indicate the distribution of TNM stage in robust, pre-frail, and frail groups; **F** reflect the grouping of frailty in the survival and death groups. Abbreviations: TNM, tumor, node and metastasis; CCI, Charlson comorbidity index

A total of 2735 lung cancer patients were identified in this study, who were diagnosed with lung cancer and received further treatment at our hospital between 2015–2018. Among which, 95 patients were removed due to incomplete baseline data in the medical record, 552 patients were excluded due to incomplete test information to calculate FI-LAB, and 421 patients were excluded because they were lost in the telephone follow-up to obtain patient survival information. The final 1667 eligible patients were included in this study (Sup. Fig. 1). Of them, 297 (17.8%) were classified as frail, most commonly patients with pre-frail status with 813 (48.8%). The mean age in the study population was 67 years old, with a greater mean age in patients with high frailty grade (Fig. 1A). Of all the patients enrolled, 65.1% (1086/1667) of patients were male, 69.1%(1152/1667) of patients with CCI score >  = 3, the numbers of patients with TNM stage I to IV were 265 (15.9%), 280 (16.8), 405 (24.3%) and 717 (43%), respectively (Sup Table 1).

**Table 1:** Univariate analysis of relationship between all-cause mortality and clinical characteristics

Character	HR (95% CI for HR)	p.value
age^a^	0.99(0.99,1.00)	0.054
gender		
female		
male	1.073(0.943,1.22)	0.284
BMI^b^		
I		
II	0.9941(0.816,1.211)	0.953
III	0.9805(0.783,1.227)	0.863
IV	0.9920(0.795,1.237)	0.943
Frailty^c^		
Robust		
Pre-frail	1.365(1.183,1.575)	< 0.001
Frail	1.616(1.349,1.936)	< 0.001
TNM stage^d^		
I		
II	1.262(0.893,1.782)	0.187
III	2.516(1.875,3.378)	< 0.001
IV	4.871(3.707,6.400)	< 0.001
Cancer histology^e^		
Adenocarcinoma		
Small cell	0.957(0.785,1.167)	0.666
Squamous cell	1.017(0.853,1.213)	0.852
Others	0.956(0.822,1.111)	0.557
Smoking history^f^		
I		
II	1.0492 0.9531 0.9076 1.213	0.516
III	0.8237 1.2140 0.6177 1.098	0.187
IV	1.1245 0.8893 0.8665 1.459	0.378
CCI^g^		
< 3		
> = 3	5.751(4.741,6.975)	< 0.001
Surgery history		
no		
yes	0.367(0.317,0.425)	< 0.001
Chemotherapy		
no		
yes	2.42(1.973,2.969)	< 0.001
Radiotherapy		
no		
yes	1.018(0.841,1.233)	0.853
Target therapy		
no		
yes	1.016(0.859,1.2)	0.855

*Abbreviations:* TNM, tumor, node and metastasis; BMI, Body Mass Index; CCI, Charlson comorbidity index;

^a^Age: median(25%,75%); ^b^BMI: Body mass index is calculated as weight in kilograms divided by height in meters squared, I: BMI < 18.5; II: 18.5 ≤ BMI < 23; III: 23 ≤ BMI < 26; IV: BMI ≥ 26; ^c^Frailty: Robust, FI-LAB < 0.2; prefrail, FI-LAB 0.2–0.35; frail, FI-LAB > 0.35; ^d^TNM stage: tumor, node and metastasis classification; ^e^Cancer histology: Tumor pathological staging is specifically divided into adenocarcinoma, squamous carcinoma, small cell lung cancer and other types of lung cancer; ^f^Smoking history: I: non-smoking (< 100 cigarettes in life-time); II: present smoking(> 10 years of smoking and > 10 cigarettes per day); III: Occasional smoking (< 10 cigarettes per month); IV: past smoking(> 10 years of smoking and > 10 cigarettes per day), has quit smoking over six months; ^g^CCI: Quantifies comorbidities based on the number and severity of diseases a patient has, and can be used to predict the risk of death from diseases

### Associations of all-cause mortality with different clinical characteristics

In this cohort study, the median follow-up time was 650 (493, 1001.5) days and 1018 patients were followed up to a mortality outcome, of which 557 (33.4%), 813 (48.8%) and 297 (17.8%) were in the robust, pre-frail and frail groups, respectively. Patients with high frailty grades had a higher median age (Fig. 1A), CCI scores were higher in patients who experienced a fatal event (Fig. 1B). In a univariate analysis, we found a higher total all-cause mortality risk in frail patients (frail vs. robust, HR = 1.616, 95% CI = (1.349,1.936, Table 1) and between 1.5–2 in model 2 to 6 in the frail group after balancing other factors. We also found higher survivability in patients with a history of prior surgery and in those without a history of chemotherapy (Fig. 1C, D). Higher all-cause mortality in patients with high TNM stage (Fig. 1E) (HR = 4.871, CI: 3.707–6.400, *p* < 0.001). Frailty scores were higher in patients who experienced a fatal event (Fig. 1F). A chi-squared test analysis of the two indicators of TNM stage and surgical history (X-squared = 904.37, *P* < 0.001) suggests that patients with low TNM stage tend to have access to surgery and a better prognosis.

### Predictive model for the primary lung cancer with frailty

**Fig. 2:**
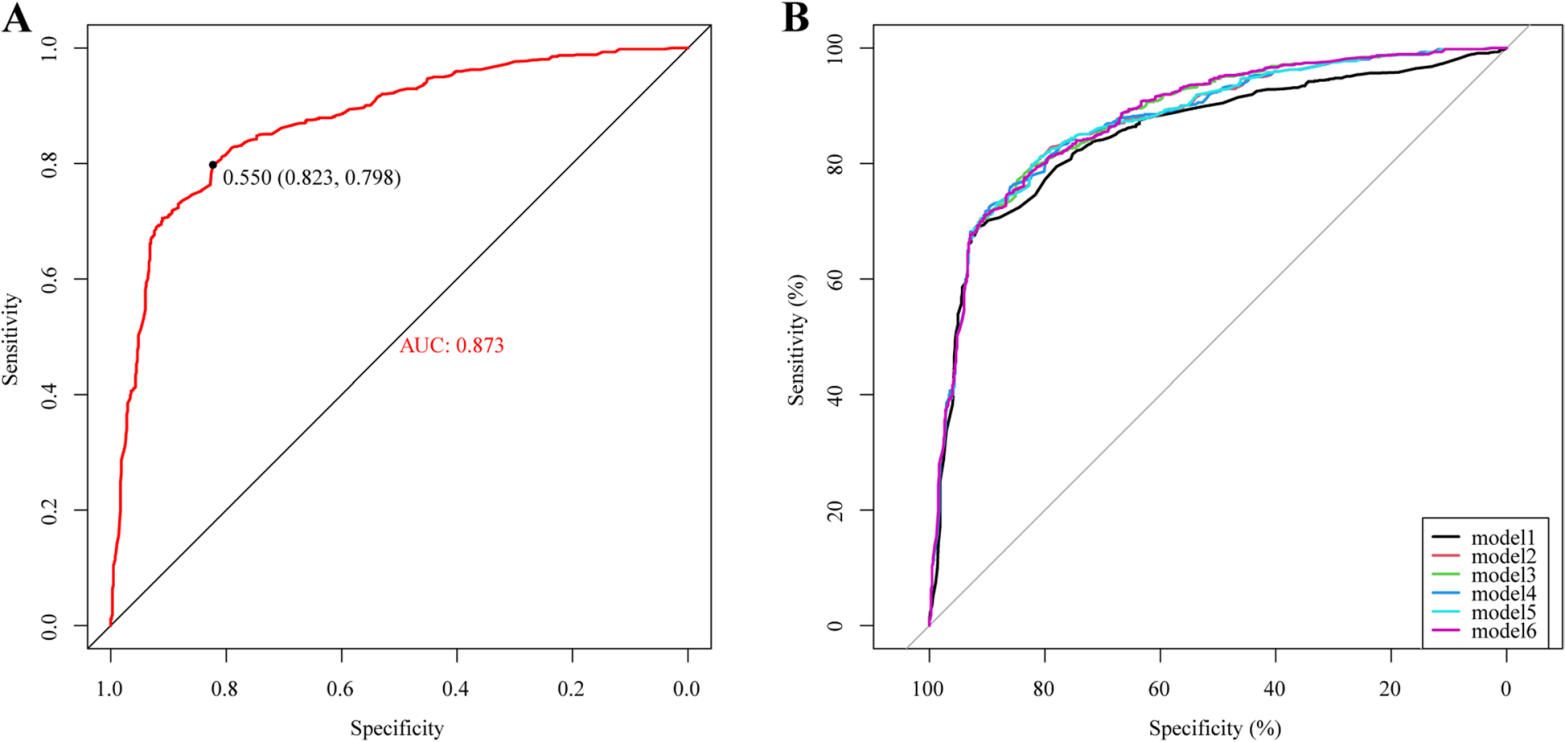
Receiver operating characteristic (ROC) curve of model 1 to 6. ROC curve to determine the optimal cut-off of multivariate analysis results in model 1 to 6; (B), model 6 was showed an AUC of 0.789

**Fig. 3:**
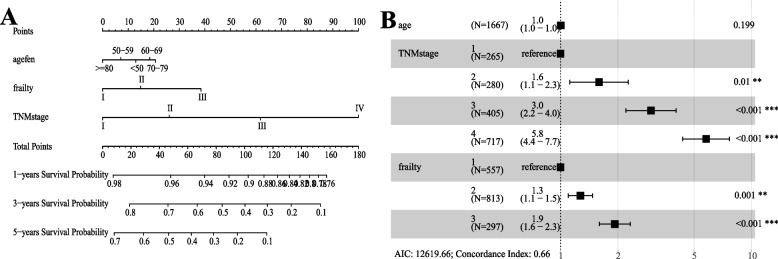
Nomogram and forest plot of model 2. **A** Nomogram for predicting the risk of all-cause mortality in model 2; **B** forest plot for predicting the risk of all-cause mortality in model 2

According to the univariate results, six indicators of age, CCI, frailty, TNMstage, surgical history, and chemotherapy history were put into the multifactorial model according to different combinations. In these six models, the model2 AUC consisting of age, frailty and TNMstage was 0.873 (Fig. 2A), and the AUC values of mod2-6 were all between 0.851–0.878 (Fig. 2B). Further comparing the predictive ability of the models: the accuracy of model 2 was 86.8% higher than that of model 1. Compared with model 2, the correlation coefficient between CCI and TNM stage reached 0.6 (Sup. Fig. 3), although the NRI value of model 3 was 0.338 (0.179,0.541), and the C-index value of model 3 was the largest compared with the rest of the matching methods, these two indicators were not suitable to be put into the model at the same time, so the final choice was Model 2 was visualized by plotting forest plots and nomogram (Fig. 3). In model 2, after balancing age and TNM stage, the risk of all-cause mortality was higher in patients with frailty status (Table 2, HR 1.9 (1.6–2.4), *P* < 0.001). Calibration plot were draw for model2 and the model results were stable (Sup. Fig. 3).

**Table 2:** Multivariate analysis of relationship between all-cause mortality and clinical characteristics

	Model1	p	Model2	p	Model3	p	Model4	p	Model5	p	Model6	p
age	1 (1–1)	< 0.001	1 (1–1)	< 0.001	1 (1–1)	< 0.001	1 (1–1)	< 0.001	1 (1–1)	0.214	1 (1–1)	< 0.001
Frailty^a^	NA	NA										
Robust	NA	NA	-	-	-	-	-	-	-	-	-	-
Pre-frail	NA	NA	1.3(1.1,1.5)	< 0.001	1.1 (1.0–1.3)	< 0.001	1.3 (1.1–1.5)	< 0.001	1.3 (1.1–1.5)	< 0.001	1.1 (1.0–1.3)	< 0.001
Frail	NA	NA	1.9 (1.6–2.4)	< 0.001	1.5 (1.2–1.8)	< 0.001	2 (1.6–2.4)	< 0.001	2 (1.7–2.4)	< 0.001	1.5 (1.2–1.8)	< 0.001
TNM stage^b^												
I	-	-	-	-	-	-	-		-		-	
II	1.3 (0.9–1.8)	< 0.001	1.5 (1.1–2.3)	< 0.001	1.7 (1.2–2.5)	< 0.001	1.5 (1.1–2.2)	< 0.001	1.3 (0.9–2)	< 0.001	1.4 (0.9–2.2)	< 0.001
III	2.5 (1.9–3.4)	< 0.001	2.9 (2.2–3.9)	< 0.001	2.3 (1.7–3.1)	< 0.001	2.8 (2–3.8)	< 0.001	2.5 (1.7–3.6)	< 0.001	1.8 (1.2–2.7)	< 0.001
IV	4.9 (3.7–6.4)	< 0.001	5.7(4.3–7.5)	< 0.001	2.4 (1.8–3.3)	< 0.001	5.1 (3.6–7.3)	< 0.001	4.7 (3.3–6.9)	< 0.001	1.9 (1.2–2.9)	< 0.001
CCI^c^	NA	NA	NA	NA			NA	NA	NA	NA		
< 3	NA	NA	NA	NA	-	-	NA	NA	NA	NA	-	-
> = 3	NA	NA	NA	NA	4.1 (3.2–5.2)	< 0.001	NA	NA	NA	NA	4.1 (3.3–5.2)	< 0.001
Surgery history	NA	NA	NA	NA	NA	NA			NA	NA		
no	NA	NA	NA	NA	NA	NA	-	-	NA	NA	-	-
Yes	NA	NA	NA	NA	NA	NA	0.9 (0.7–1.1)	< 0.001	NA	NA	0.9(0.7–1.2)	< 0.001
Chemotherapy	NA	NA	NA	NA	NA	NA	NA	NA				
no	NA	NA	NA	NA	NA	NA	NA	NA	-	-	-	-
Yes	NA	NA	NA	NA	NA	NA	NA	NA	1.2 (0.9–1.6)	0.164	1.2 (0.9–1.7)	< 0.001

*Abbreviations:* *TNM* tumor, node and metastasis, *CCI* Charlson comorbidity index

^a^Frailty: Robust, FI-LAB < 0.2; prefrail, FI-LAB 0.2–0.35; frail, FI-LAB > 0.35; ^b^TNM stage: tumor, node and metastasis classification; ^c^CCI: Quantifies comorbidities based on the number and severity of diseases a patient has, and can be used to predict the risk of death from disease

## Discussion

In this cohort study of primary lung cancer which included 1667 patients, we investigated the relationship between FI-LAB calculated based on laboratory data and all-cause mortality in patients. This study provided important evidence for the use of FI-LAB in lung cancer patients in Chinese. This study showed that baseline frailty in lung cancer patients was significantly associated with overall survival(OS). Frailty was an independent predictor of OS in lung cancer patients, and other characteristics also played an important role.

Appropriate assessment methods facilitate early screening of lung cancer patients for frailty. However, to date, there is no standard method for assessing the degree of frailty in patients with lung cancer. The 2019 International Conference on Frailty and Sarcopenia Research (ICFSR) recommendations summarized the five most commonly used methods for the assessment of FI, which was CSF, FI-LAB, frailty phenotype, frail scale, EFS [17–20]. However, these methods had disadvantages such as high subjectivity and poor data availability, making it difficult to obtain widespread dissemination. The ICFSR guidelines strongly recommend screening for all adults aged 65 years and older. The next step is to make clinical judgment on high-risk patients [26]. With the popularity of hospital electronic medical records, simplifying the difficulty of FI data collection, the use of FI-LAB to assess the frailty index in lung cancer patients is considered convenient and feasible [27].

To our knowledge this is the first large cohort study of FI-LAB validated in the lung cancer population. The study assessed FI by FI-LAB developed at the West China Hospital^25^, which was specified for patients undergoing chemotherapy for primary lung cancer. Previous studies had shown that frailty is very common in oncology patients and was associated with the development of complications, reduced OS, and increased all-cause mortality [2, 11, 12, 14, 25, 28]. Our study also supports the identification of patients with high grade of frailty by early identification. Lippi L et al. suggested that pulmonary complications can be effectively prevented, even the high risk of frailty can be reversed by effective rehabilitation management [29]. Due to the development of medical specialization, many frailty patients do not receive attention and do not complete rehabilitation programs [30]. Interdisciplinary care is an effective solution to address this problem [29]. This new model promotes rehabilitation programs for frailty patients between inpatients and outpatients, which can effectively bridge the gap between outpatient and inpatient medical conditions.

In our study, in addition to frailty, other associated factors negatively affected survival: patients with high TNM stage and a history of chronic disease had a higher risk of all-cause mortality. A study of elderly patients with early-stage non-small cell lung cancer confirmed that frailty was associated with reduced OS and that patients with frailty survived an average of 2.5 years and were more likely to die from causes unrelated to their primary diseases [31]. Patients with frailty may have more comorbidities, such as hypertension and diabetes. It may also lead to a poorer clinical outcome in patients with CCI greater than 3 and a long history of previous smoking. CCI is a commonly used index to summarize comorbidities. First proposed in 1987, CCI is a proven method for classifying comorbidities that may alter a patient's risk of death [32]. Comorbidities are often associated with poor prognosis in cancer patients [33]. An acute leukemia study showed that overall survival (OS) in patients with CCI < 3 was twice that in patients with CCI ≥ 3 [34]. This study also found the same conclusion in a cohort of lung cancer patients.

The study has some limitations: first, the study was done in a single medical institution with a limited sample size, and some bias is inevitable. Since this study was conducted only in lung cancer patients, there was no evidence for generalization to the tumor patients. In addition, there is no consensus on the specific inclusion of indicators using cumulative deficits and rates of the total number of variables considered [25, 35, 36]. The exact scale to be used for promotion in future clinical practice needs to be explored in further prospective clinical studies.

## Conclusions

The FI-LAB frailty grade calculated based on routine laboratory tests has a high predictive value for all-cause mortality in patients with primary lung cancer, and the higher the frailty grade, the greater the risk of all-cause mortality. The use of FI-LAB to characterize frailty prior to treatment helps guide decision-making and patient counseling. In the context of widespread electronic medical records in hospitals, it is convenient and feasible to use FI-LAB to assess the prognosis of tumor patients.

## Supplementary Information

**Additional file 1.**

**Additional file 2.**

## Data Availability

The datasets used and/or analyzed during the current study are available from the corresponding author on reasonable request.
