# Supplementary material for: Prognostic significance of frailty status in patients with primary lung cancer
Source: BMC Geriatr. 2023 Jan 25;23:46. doi: 10.1186/s12877-023-03765-w (PMC9878966; doi:10.1186/s12877-023-03765-w)
Supplement: Supplementary file 1 — Additional file 1. [file 12877_2023_3765_MOESM1_ESM.docx]

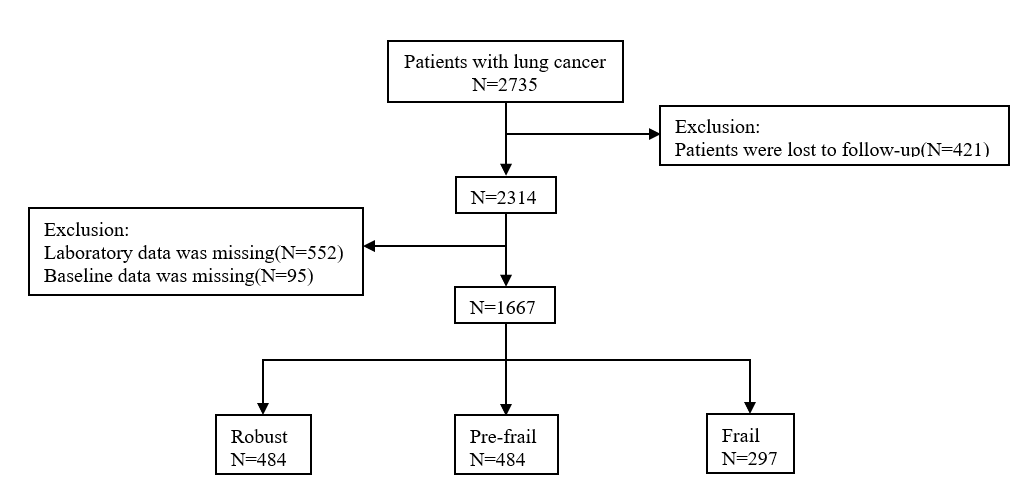


**Supplement Figure 1：Flowchart of the subjects selection process.**


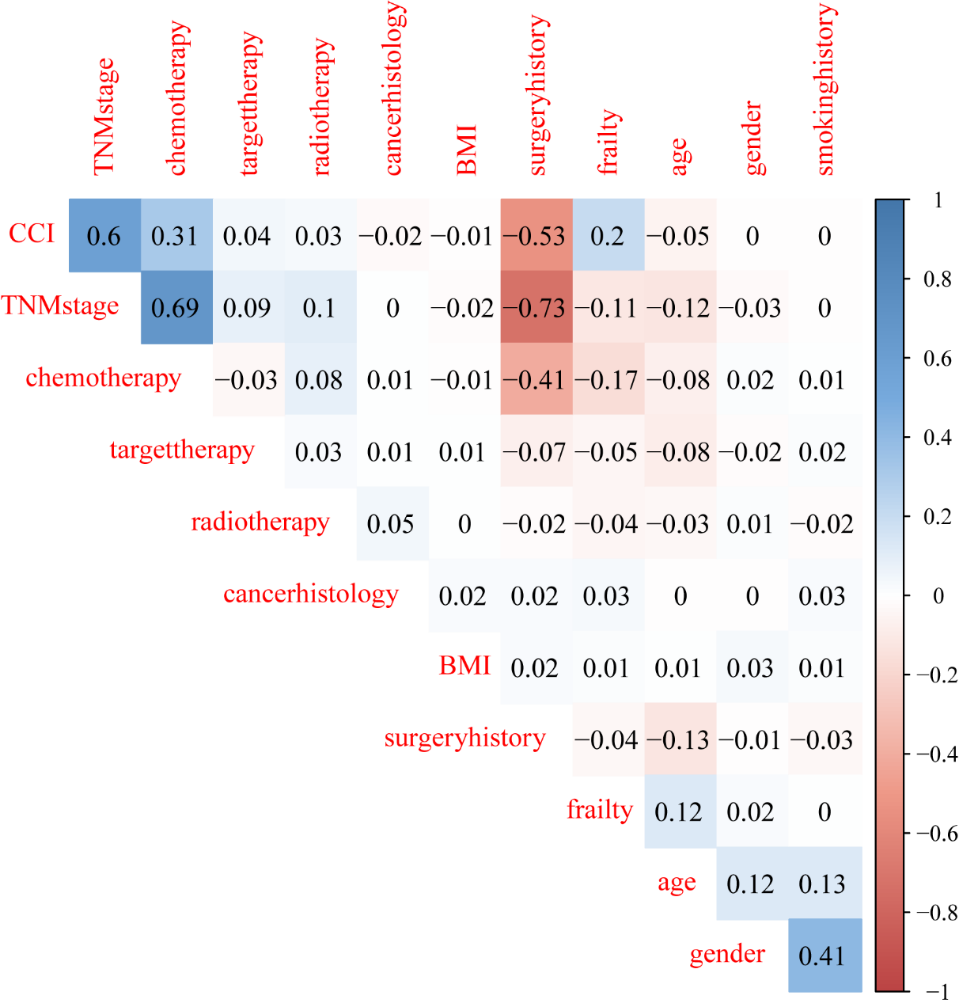


**Supplement Figure 2：Correlation analysis of all variables**


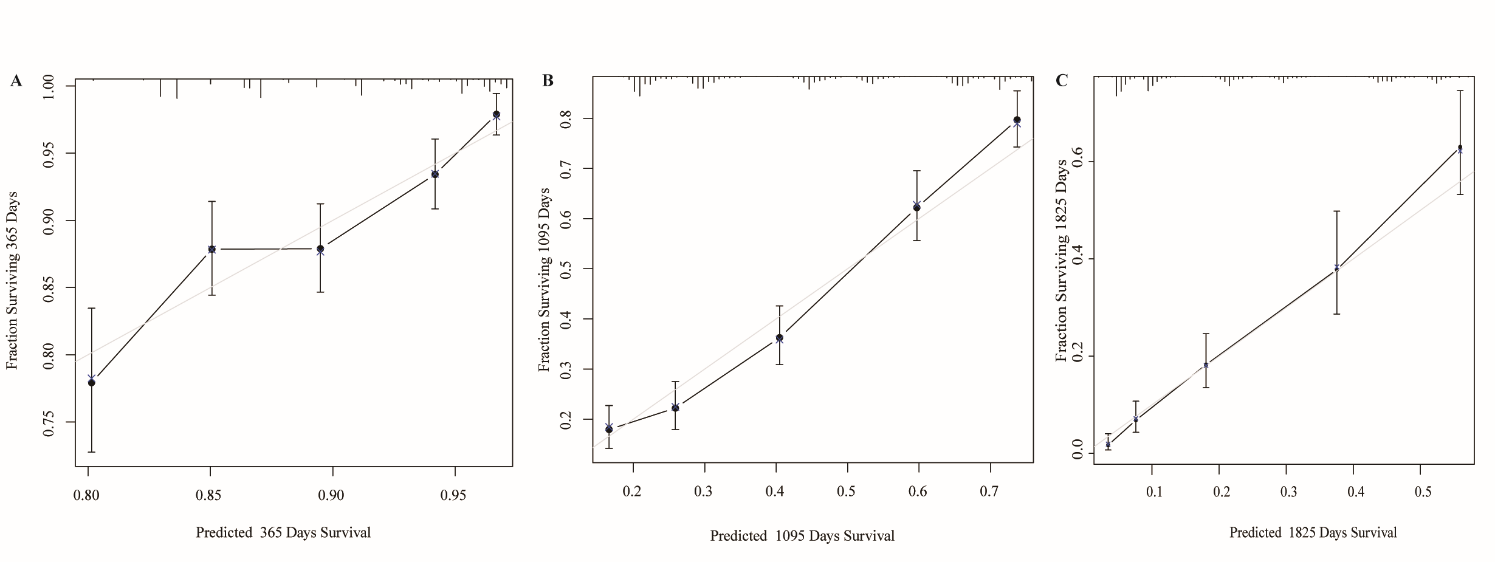


**Supplement Figure 3：Calibration plot for 1, 3 and 5 years**
