# Supplementary material for: Prognostic significance of frailty status in patients with primary lung cancer
Source: BMC Geriatr. 2023 Jan 25;23:46. doi: 10.1186/s12877-023-03765-w (PMC9878966; doi:10.1186/s12877-023-03765-w)
Supplement: Supplementary file 2 — Additional file 2. [file 12877_2023_3765_MOESM2_ESM.docx]

**Supplement Table1. Baseline charcateristics of patients with lung cancer**

|  | **robust**  (N=557,33.4%) | **pre-frail**  (N=813, 48.8%) | **frail**  (N=297, 17.8%) | **Overall**  (N=1667) |
| --- | --- | --- | --- | --- |
| Age | 66 (60,69) | 67 (61,70) | 68 (64,72) | 67(61,70) |
| gender |  |  |  |  |
| female | 194 (34.8%) | 299 (36.8%) | 88 (29.6%) | 581 (34.9%) |
| male | 363 (65.2%) | 514 (63.2%) | 209 (70.4%) | 1086 (65.1%) |
| Cancer histology^a^ |  |  |  |  |
| adenocarcinoma | 254 (45.6%) | 361 (44.4%) | 118 (39.7%) | 733 (44.0%) |
| small cell | 66 (11.8%) | 99 (12.2%) | 36 (12.1%) | 201 (12.1%) |
| squamous cell | 83 (14.9%) | 124 (15.3%) | 60 (20.2%) | 267 (16.0%) |
| others | 154 (27.6%) | 229 (28.2%) | 83 (27.9%) | 466 (28.0%) |
| TNM stage^b^ |  |  |  |  |
| I | 55 (9.9%) | 100 (12.3%) | 110 (37.0%) | 265 (15.9%) |
| II | 142 (25.5%) | 111 (13.7%) | 27 (9.1%) | 280 (16.8%) |
| III | 125 (22.4%) | 225 (27.7%) | 55 (18.5%) | 405 (24.3%) |
| IV | 235 (42.2%) | 377 (46.4%) | 105 (35.4%) | 717 (43.0%) |
| Smoking history^c^ |  |  |  |  |
| no | 376 (67.5%) | 573 (70.5%) | 194 (65.3%) | 1143 (68.6%) |
| yes | 181 (32.5%) | 240 (29.5%) | 103 (34.7%) | 524 (31.4%) |
| BMI^d^ |  |  |  |  |
| <18.5 | 68 (12.2%) | 91 (11.2%) | 33 (11.1%) | 192 (11.5%) |
| 18.5-23 | 270 (48.5%) | 396 (48.7%) | 145 (48.8%) | 811 (48.7%) |
| 23-26 | 105 (18.9%) | 162 (19.9%) | 54 (18.2%) | 321 (19.3%) |
| >26 | 114 (20.5%) | 164 (20.2%) | 65 (21.9%) | 343 (20.6%) |
| radiotherapy |  |  |  |  |
| no | 482 (86.5%) | 712 (87.6%) | 270 (90.9%) | 1464 (87.8%) |
| yes | 75 (13.5%) | 101 (12.4%) | 27 (9.1%) | 203 (12.2%) |
| CCI^e^ |  |  |  |  |
| <3 | 251 (45.1%) | 196 (24.1%) | 68 (22.9%) | 515 (30.9%) |
| >=3 | 306 (54.9%) | 617 (75.9%) | 229 (77.1%) | 1152 (69.1%) |
| Surgery history |  |  |  |  |
| no | 283(50.8%) | 500(61.5%) | 153(51.5%) | 936(56.1%) |
| yes | 274(49.2%) | 313(38.5%) | 144(48.5%) | 731(43.9%) |
| Chemotherapy |  |  |  |  |
| no | 88 (15.8%) | 127 (15.6%) | 122 (41.1%) | 337 (20.2%) |
| yes | 469 (84.2%) | 686 (84.4%) | 175 (58.9%) | 1330 (79.8%) |
| Target therapy |  |  |  |  |
| no | 457 (82.0%) | 716 (88.1%) | 252 (84.8%) | 1425 (85.5%) |
| yes | 100 (18.0%) | 97 (11.9%) | 45 (15.2%) | 242 (14.5%) |

Abbreviations: TNM, tumor node metastasis; BMI, Body Mass Index; CCI, Charlson comorbidity index;

^a^Cancer histology: Tumor pathological staging is specifically divided into adenocarcinoma, squamous carcinoma, small cell lung cancer and other types of lung cancer; ^b^TNM stage: Tumor, node and metastasis classification; ^c^Smoking history: no, previous history of smoking or occasional smoking; yes, >10 years of smoking and >10 cigarettes per day; ^d^BMI: Body mass index is calculated as weight in kilograms divided by height in meters squared; ^e^CCI: Quantifies comorbidities based on the number and severity of diseases a patient has, and can be used to predict the risk of death from diseases.

**Supplement Table2. AUC and Cindex values for model 1- 6**

|  | Model 1 | Model 2 | Model 3 | Model 4 | Model 5 | Model 6 |
| --- | --- | --- | --- | --- | --- | --- |
|  | 0.555(0.917,0.690) | 0.550(0.823,0.798) | 0.598(0.847,0.771) | 0.580(0.897,0.724) | 0.550(0.823,0.797) | 0.633(0.903,0.711) |
| AUC | 0.851 | 0.873 | 0.878 | 0.873 | 0.873 | 0.878 |
| C-index | 0.645 | 0.655 | 0.677 | 0.657 | 0.656 | 0.678 |

**Supplement Table3. NRI values for comparison between models**

|  | M1 vs. M2 | M2 vs. M3 | M2 vs. M4 | M2 vs. M5 | M2 vs. M6 |
| --- | --- | --- | --- | --- | --- |
| NRI | 0.868(0.588,1.023) | 0.357(0.158,0.572) | 0(-0.060,0.046) | 0 (-0.068,0.127) | 0.325(0.170,0.641) |
